# 17beta-hydroxysteroid dehydrogenase type 1 modulates breast cancer protein profile and impacts cell migration

**DOI:** 10.1186/bcr3207

**Published:** 2012-06-12

**Authors:** Juliette A Aka, Mouna Zerradi, François Houle, Jacques Huot, Sheng-Xiang Lin

**Affiliations:** 1 Laboratory of Molecular Endocrinology and Oncology, Centre Hospitalier Universitaire de Québec Research Center (CHUQ - CHUL) and Department of Molecular Medicine, Laval University, 2705 boulevard Laurier, Québec G1V 4G2, Canada; 2 Cancer Research Center of Laval University and Centre Hospitalier Universitaire de Québec Research Center (CHUQ - L'Hôtel-Dieu de Québec), 9 rue McMahon, Québec G1R 2J6, Canada

## Abstract

**Introduction:**

Human 17beta-hydroxysteroid dehydrogenase type 1 (17β-HSD1) is a steroid-converting enzyme that has long been known to play critical roles in estradiol synthesis and more recently in dihydrotestosterone (DHT) inactivation, showing a dual function that promotes breast cancer cell proliferation. Previously, we reported the first observation of the influence of the enzyme on endogenous estrogen-responsive gene expression. Here, we demonstrate the impact of 17β-HSD1 expression on the breast cancer cell proteome and investigate its role in cell migration.

**Methods:**

17β-HSD1 was stably transfected in MCF7 cells and the proteome of the generated cells overexpressing 17β-HSD1 (MCF7-17βHSD1 cells) was compared to that of the wild type MCF7 cells. Proteomics study was performed using two-dimensional gel electrophoresis followed by mass spectrometry analysis of differentially expressed protein spots. Reverse transcription quantitative real-time PCR (RT-qPCR) was used to investigate the transcription of individual gene. The effect of 17β-HSD1 on MCF7 cell migration was verified by a wound-healing assay.

**Results:**

Proteomic data demonstrate that the expression of more than 59 proteins is modulated following 17β-HSD1 overexpression. 17β-HSD1 regulates the expression of important genes and proteins that are relevant to cell growth control, such as BRCA2 and CDKN1A interacting protein (BCCIP) and proliferating cell nuclear antigen (PCNA) which are down- and upregulated in MCF7-17βHSD1 cells, respectively. RT-qPCR data reveal that 17β-HSD1 increases the mRNA levels of estrogen receptors (ER) alpha and beta by 171 and 120%, respectively, while decreasing that of the androgen receptor by 64%. Interestingly, 17β-HSD1 increases the mRNA transcript (by 3.6 times) and the protein expression of the metastasis suppressor gene nm23-H1 and the expression of the two enzymes are closely correlated. We have further shown that 17β-HSD1 expression is associated with an increase of MCF7 cell migration.

**Conclusions:**

In addition to the regulation of important genes, we have demonstrated for the first time that 17β-HSD1 increases breast cancer cell migration, in spite of its positive regulation of the antimetastatic gene *NM23*. This is also correlated to its stimulation of breast cancer cell growth, further confirming its targeting in ER positive breast cancer. The novel findings in this study suggest several directions for future research on the contribution of 17β-HSD1 to breast cancer progression and related treatment.

## Introduction

Breast cancer is the most frequent cancer affecting women. The malignancy accounts for about one in ten cancers in the world and is diagnosed in one million women each year [1,2]. In North America, breast cancer is the second most important cause of death from cancer in women, after lung cancer, and the leading cause of cancer death among those between 20 and 59 years of age [3,4]. After increasing through the 80s and 90s, breast cancer incidence rates showed a welcome decrease of 3.5% per year from 2001 to 2004 and the mortality rate decreased by 1.9% per year in the United States between 1998 and 2006 [3,5]. This reflects an improvement in the diagnosis and treatment of the disease, yet it remains of prime importance.

Epidemiological evidence indicates that most breast cancer risk factors are associated with prolonged exposure of the mammary gland to high levels of estradiol (E2). This potent estrogen plays a crucial role in the development and evolution of hormone-dependent breast cancer [6]. About 60% of premenopausal and 75% of postmenopausal breast cancer patients show a hormone dependency [7]. The final steps of E2 biosynthesis implicate two principal pathways in breast cancer tissue: the aromatase pathway transforms androgens into estrogens, and the sulfatase pathway converts the inactive hormones estrone sulfate (E1S) and dehydroepiandrosterone sulfate (DHEA-S) into estrone (E1) and dehydroepiandrosterone (DHEA), respectively, via the action of steroid sulfatase (STS). Different enzymes further convert DHEA to 5α-androstene-3β,17β-diol (A-diol) and the latter to testosterone which can in turn be converted to E2 by aromatase. The inactive E1, synthesized by both STS and aromatase, is converted to the potent E2 by the action of reductive 17beta-hydroxysteroid dehydrogenases (17β-HSDs) [8-10]. In breast cancer cells, E2 is principally synthesized by 17β-HSD type 1 (17β-HSD1), with the reduced form of nicotinamide adenine dinucleotide phosphate (NADPH) as a cofactor. Previously, we reported the dual function of 17β-HSD1 in estradiol synthesis and dihydrotestosterone (DHT) inactivation stimulating cell proliferation [11]. Analyses of 17β-HSD1 mRNA expression in breast carcinoma specimens from patients revealed that high expression of the enzyme correlates with a weak prognosis for breast cancer [12-14]. Despite these observations, the relationship between 17β-HSD1 expression and that of genes and proteins involved in breast cancer cell growth has not been established. The aim of the present study was to investigate the impact of 17β-HSD1 overexpression on the protein profile of breast cancer cells. The MCF7 cell line is a human hormone-dependent breast cancer cell line widely used for breast cancer studies that expresses both estrogen and androgen receptors. Since the cell line barely expresses endogenous 17β-HSD1 [11,15], we used it as cell model for the increase of 17β-HSD1 expression. The proteomic approach using two-dimensional gel electrophoresis is the most popular tool to study global changes in protein profile following biological or chemical treatments. We thus used this technique to analyse the proteomic modification of MCF7 cells in response to 17β-HSD1 overexpression. Following the proteomics analysis, reverse transcription quantitative real-time PCR (RT-qPCR) was used to investigate the gene transcription of a number of differentially expressed proteins, such as proliferating cell nuclear antigen (PCNA) and the metastasis suppressor gene nm23-H1. The overexpression experiments, combined with further siRNA knockdown analysis, demonstrated a strong positive correlation between nm23-H1 regulation and 17β-HSD1 expression. We thus hypothesized that 17β-HSD1 could be implicated in breast cancer cell metastasis and evaluated its effect on MCF7 cell migration.

## Materials and methods

### Cell culture and generation of stably-transfected MCF7-17βHSD1 cells

Wild type (WT) MCF7 and T47D cells were cultured as previously described, with MCF7 cell culture medium containing 1 nM β-E2 [11]. Recombinant plasmid containing 17β-HSD1 cDNA and the 17β-HSD1 stably transfected-MCF7 cells (MCF7-17βHSD1 cells) were generated as previously described [11].

### Generation of protein extracts for proteomics analysis

WT MCF7 and MCF7-17βHSD1 cells were defrosted at the same time and cultured in flasks (75 cm^2 ^growth area) in complete medium containing β-E2. After three passages, cells were seeded into 10 cm diameter dishes and cultured until reaching the desired confluence. For protein sample preparation, cells having reached 80 to 90% confluence were washed twice with cold phosphate-buffered saline (PBS) and scraped with a rubber policeman in 1.2 mL PBS. Cells were collected in eppendorf tubes (Eppendorf, Mississauga, Ontario, Canada) and centrifuged at 3000 rpm for 5 minutes. The cell pellets were re-suspended in 500 μL lysis buffer T8 (7 M urea, 2 M thiourea, 3% CHAPS, 20 mM DTT, 5 mM TCEP, 0.5% IPG buffer pH 4-7, 0.25% IPG buffer pH 3-10) containing 50 mM tris-HCl pH 8.8, 1 mM phenylmethylsulfonyl fluoride (PMSF) and 1% protease inhibitors cocktail (EMD Chemicals, Gibbs-town, NJ, USA). Protein samples were precipitated using the two-dimensional Clean-Up Kit (GE Healthcare, Piscataway, NJ, USA) and resolubilized in T8 buffer. The protein samples included three independent biological replicates (coming from three independent cell culture experiments), representing total proteins from each cell line (MCF7 and MCF7-17βHSD1) for a total of six samples. The protein concentrations were determined using the two-dimensional Quant Kit (GE Healthcare, Piscataway, NJ, USA).

### Two-dimensional gel electrophoresis

For the first dimension, 200 µg total protein samples from MCF7 and MCF7-17βHSD1 cells were loaded onto 24-cm pH 4-7 immobilized pH gradient (IPG) strips (Immobiline DryStrips; GE Healthcare). Strips were rehydrated for 10 hours at 30 volts and isoelectric focusing was performed on an IPGphorII IEF system (GE Healthcare). For the second-dimension sodium dodecyl sulphate-polyacrylamide gel electrophoresis (SDS-PAGE), focused Immobiline DryStrips were equilibrated twice for 15 minutes in an equilibration buffer (50 mM tris-HCl pH 8.8, 6 M urea, 30% glycerol, 2% SDS, trace of bromophenol blue) containing 10 mg/mL DTT for the first equilibration and 25 mg/mL iodoacetamide for the second one. Immobiline DryStrips were then transferred onto the surface of a 12% acrylamide gel (20 × 25 × 0.1 cm) and sealed using 0.5% agarose. Gels were run in an Ettan DALT*twelse *system (GE Healthcare) in a standard tris-glycine SDS-PAGE buffer at 40 mA/gel and 15^o^C until the tracking dye reached the end of the gel. Three independent protein samples coming from three independent cell culture experiments were run for each cell line. Gels were fixed overnight in 40% methanol, 7% acetic acid, stained with Sypro Ruby (Invitrogen, Burlington, Ontario, Canada) and scanned with the ProXpress CCD scanner (PerkinElmer, Waltham, MA, USA). The two-dimensional gel electrophoresis was performed on the Proteomic Platform of the Infectious Disease Research Center (Québec, Canada).

### Two-dimensional gel image analysis

Protein spot detection, spot matching and semiquantitative statistical analysis were performed using the Progenesis software version PG240 (Nonlinear Dynamics, Durham, NC, USA). For each cell line, three different gel images were analyzed and a corresponding synthetic image reference was obtained. After computer matching, detected spots and spot matches were manually edited for better accuracy. A spot had to be present in at least two of the three replicate gels to be considered in the analysis. The detection of protein spots differentially expressed was performed using the *t*-test (*P *< 0.05) and the Intelligent Noise Correction Algorithm (INCA) volume and proteins that were differentially expressed two-fold or higher were considered significant. Eighteen protein spots were selected among the differentially expressed spots and were excised from Sypro Ruby-stained two-dimensional gels using a ProXcision robot (PerkinElmer, Waltham, MA, USA) and sent for mass spectrometry (MS) analysis.

### Mass spectrometry and protein identification

MS experiments were performed by the Proteomics Platform of the Eastern Quebec Genomics Center (Québec, Canada). Protein spots were washed with water and tryptic digestion was performed on a MassPrep liquid-handling robot (Waters, Milford, MA, USA) according to the manufacturer's specifications and the protocol of Shevchenko *et al*. [16], with the modifications suggested by Havlis *et al*. [17]. Peptide samples (aliquots of the digested protein samples) were separated by online reversed-phase (RP) nanoscale capillary liquid chromatography (nano LC) and analyzed by electrospray tandem mass spectrometry (ES-MS/MS). The experiments were performed with a Thermo Surveyor MS pump connected to an LTQ linear ion trap mass spectrometer (Thermo Fisher Scientific, San Jose, CA, USA) equipped with a nanoelectrospray ion source (Thermo Fisher Scientific). Peptide separation took place on a PicoFrit column BioBasic C18, 10 cm × 0.075 mm internal diameter (New Objective, Woburn, MA, USA) with a linear gradient from 2 to 50% solvent B (acetonitrile, 0.1% formic acid) in 30 minutes, at 200 nL/min (obtained by flow-splitting). Mass spectra were acquired using a data dependent acquisition mode using Xcalibur software version 2.0 (Thermo Fisher Scientific). Each full scan mass spectrum (400 to 2000 m/z) was followed by collision-induced dissociation of the seven most intense ions. The dynamic exclusion (30 seconds exclusion duration) function was enabled, and the relative collisional fragmentation energy was set to 35%.

All MS/MS samples were analyzed using the Mascot algorithm (Matrix Science, London, UK; version Mascot) and the Uniref100_14_0_Homo_sapiens_9606 database (version with 89892 entries). Mascot was searched with a fragment ion mass tolerance of 0.50 Da and a parent ion tolerance of 2.0 Da. Iodoacetamide derivative of cysteine was specified as a fixed modification and oxidation of methionine was specified as a variable modification. Two missed cleavages were allowed.

Scaffold (version Scaffold_2_01_02, Proteome Software Inc., Portland, OR, USA) was used to validate MS/MS-based peptide and protein identifications. The protein identification cut off was set at a confidence level of 95% (Mascot score > 33) with at least two peptides matching to a protein. Proteins that contained similar peptides and could not be differentiated based on MS/MS analysis alone were grouped to satisfy the principles of parsimony.

### Reverse transcription quantitative real-time PCR and semiquantitative RT-PCR

Total RNA was isolated from cells using Trizol Reagent (Invitrogen, Burlington, Ontario, Canada) in 6-well plates and treated with DNase 1. Analysis of the RNA integrities using the Bioanalyzer 2100 (Agilent Technologies, Mississauga, Ontario, Canada) and the RNA 6000 Nano Chips (Agilent Technologies, Mississauga, Ontario, Canada) showed good qualities for all the RNA samples with RNA integrity numbers (RIN) higher than 8/10. RNA samples for RT-qPCR analyses comprised two biological repetitions for each condition and cell line. For each sample, mRNA quantifications were performed twice as previously described [11,18] with *Atp5o*, *Hprt1 *and *G6PD *genes used as internal controls. The procedures were performed at the Q_RTPCR Platform service at CHUQ-CHUL Research Center (Quebec, Canada). The primers used for the amplification and the corresponding cDNA fragments of each mRNA are shown in Additional file 1. The mRNA levels were expressed as mRNA copies/µg total RNA.

Semiquantitative RT-PCR was carried out and analyzed as previously described [11] except for the number of cycles, which was 30.

### siRNA synthesis and transfection

The sense and antisense sequences of three 17β-HSD1 siRNAs were selected and synthesized as previously described [11]. Transfection of T47D cells with siRNA was carried out in 6-well plates using Lipofectamine siRNAMax (Invitrogen), 3x10^5 ^cells/well and 200 nM mixed 17β-HSD1-specific siRNAs.

For cell migration assays, MCF7-17βHSD1 cells were transfected with 100 nM mixed 17β-HSD1-specific siRNAs in 3.5 cm diameter dishes. Control cells were transfected with scramble (control) siRNA [11].

### Cell migration assay

Cell migration was evaluated by using a wound-healing assay. First, MCF7 and MCF7-17βHSD1 cells were cultivated in 75 cm^2 ^culture flasks in complete growth medium. Cells, at low passage number, were seeded at high density into 3.5 cm diameter dishes in E2-free medium containing 5% fetal bovine serum (FBS). Two days later, straight scratches were made in triplicate across confluent monolayer cultures using a p200 micropipette tip. Thereafter, cells were washed five times with fresh E2-free medium and were incubated in the same medium. Second, MCF7-17βHSD1 cells were transfected with 17β-HSD1-specific siRNAs or scramble siRNA (control siRNA) in 3.5 cm diameter dishes in complete growth medium, and were incubated. Forty-eight hours after transfection, a wound was created by manually scraping the cell monolayer as described above. Cells were then washed five times and incubated in E2-free medium containing 5% FBS. All experiments were done in quadruplicate. The movements of cells in the scratched area were monitored by capturing images every 15 minutes for a total duration of 48 hours using the ×10 objective lens of a phase-contrast microscope. The scratch widths were measured at specific time points using the NIH ImageJ software.

### Western blot

WT MCF7 and MCF7-17βHSD1 cells were cultured in complete medium containing β-E2 and total proteins were extracted from cells with complete T8 lysis buffer. Equal volumes of proteins were separated by 12% SDS-PAGE and then electro-blotted onto nitrocellulose membranes. The membranes were blocked with 5% non-fat milk in PBS-tween 20 (PBS-T) for one hour at room temperature. After blocking, the membranes were incubated for two hours at room temperature in 5% non-fat milk in PBS-T containing the following primary antibodies against the indicated proteins: 17β-HSD1 (1:100,000 dilution of ab51045) from Abcam (Cambridge, MA, USA), PCNA (1:500 dilution of sc-7907), nm23-H1 (1:500 dilution of sc-343), BCCIP (1:300 dilution of SC-130898) from Santa Cruz Biotechnology (Santa Cruz, CA, USA), and β-actin as the internal control (1:7,500 dilution of a monoclonal antibody, from Sigma). Next, membranes were incubated for one hour at room temperature with a horseradish peroxidase-conjugated secondary antibody (Santa Cruz Biotechnology) diluted 10,000 times. Protein signals were visualized with Chemiluminescence Reagent (PerkinElmer) and bands were quantified using the NIH ImageJ software. The ratios between the signals of the protein of interest and β-actin were calculated to determine the relative protein expression values.

## Results

### Overexpression of 17β-HSD1 modulates the protein profile of MCF7 cells

**Figure 1:**
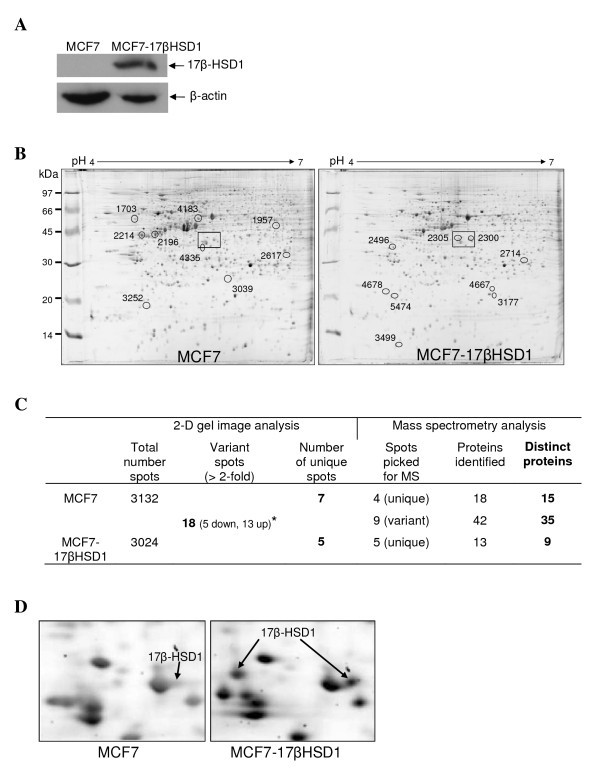
**Proteomic analysis of wild type (WT) MCF7 cells and MCF7 cells stably transfected with 17β-HSD1 (MCF7-17βHSD1)**. **(A) **17beta-hydroxysteroid dehydrogenase type 1 (**17β**-HSD1) and β-actin expression between WT MCF7 and MCF7-17βHSD1 revealed by western blots. **(B) **Representative two-dimensional gel images for WT MCF7 and MCF7-17βHSD1 cells. Whole cell lysates (200 µg) from each cell were separated by two-dimensional electrophoresis and visualized by Sypro Ruby staining. The two-dimensional gels were scanned and the differentially expressed (2-fold or higher, *P *< 0.05) proteins were detected using Progenesis software. The 18 differentially expressed protein spots that were selected for mass spectrometry (MS) analysis are marked with circles. Protein spots upregulated in MCF7-17βHSD1 are depicted in the MCF7-17βHSD1 proteome image; protein spots downregulated in MCF7-17βHSD1 are depicted in the MCF7 proteome image. The numbers refer to the spot number listed in Table 1 and Additional file 2. The squares represent the indicated area shown in more detail in **(D)**. **(C) **Summary of the numbers of spots and proteins obtained from the proteomics data. *Upregulated (up) and downregulated (down) proteins in MCF7-17βHSD1 as compared to WT MCF7 cells. **(D) **Zoom showing some differentially expressed protein spots from WT MCF7 and MCF7-17βHSD1 comparison. Arrows indicate 17β-HSD1 protein which was revealed by MS analysis to be present in the spot numbers 2,305 (unique to MCF7-17βHSD1) and 2,300 (upregulated in MCF7-17βHSD1 as compared to WT MCF7 cells).

To investigate the proteomic modifications of MCF7 cells in response to 17β-HSD1 overexpression, we performed two-dimensional gel analysis using total protein lysates of the WT breast cancer cell line MCF7 and MCF7 cells overexpressing 17β-HSD1 cultured in medium containing E2. We then compared the proteomic profile of the two cell lines. To do so, we first stably transfected WT MCF7 cells with a 17β-HSD1 plasmid to generate the 17β-HSD1 stably transfected cells (MCF7-17βHSD1 cells). Western blot showed an increase in 17β-HSD1 expression in the MCF7-17βHSD1 cells (Figure 1A). Proteomic analyses were carried out on six two-dimensional electrophoresis gels made from three independent biological repetitions of protein samples from MCF7-17βHSD1 cells and the parent cells. The two cell lines displayed similar spot patterns (Figure 1B) which allowed a good spot alignment for the proteome comparison. MCF7-17βHSD1 protein samples exhibited a lower number of protein spots (3,024) than MCF7 (3,132 spots). The proteomic analyses using the Progenesis software and a *t*-test (with a *P-*value < 0.05) identified 30 significant differential protein spots between MCF7 and MCF7-17βHSD1 as follows: 5 spots downregulated and 13 spots upregulated in MCF7-17βHSD1 as compared to MCF7, for a total of 18 spots that varied 2-fold or more, while 7 and 5 spots were unique to MCF7 and MCF7-17βHSD1 samples, respectively (Figure 1C).

The analyses by MS of 18 protein spots (Figure 1B), selected among the differentially expressed spots, allowed the identification of proteins with a known UniProt accession number among all the spots for a total of 73 proteins. The numbers of proteins found in each cell line are listed in Figure 1C. Some spots contained more than one protein and some proteins were present in more than one spot. For example, 17β-HSD1 was identified in the spot numbers 2,300 and 2,305 (Figure 1B and 1D). This resulted in the identification of 59 distinct proteins distributed as follows: 15 and 9 proteins from spots unique to MCF7 and MCF7-17βHSD1 respectively, and 35 proteins from spots upregulated in either cell line. These results showed that 17β-HSD1 modulates protein profile in MCF7 cells.

**Figure 2:**
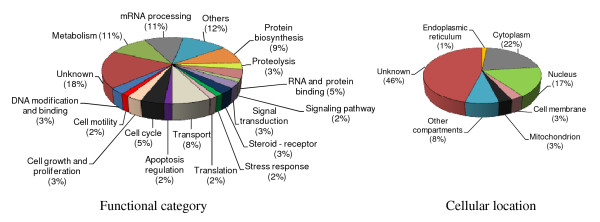
**Functions and cellular locations of the differentially expressed proteins between wild type MCF7 cells and MCF7 cells stably transfected with 17β-HSD1 (MCF7-17βHSD1)**. The Uniprot database [19] was used to generate the cellular location and the molecular function and/or biological process of each of the 59 nonredundant (distinct) proteins identified by mass spectrometry analysis as differentially regulated. Of the 59 distinct proteins, the percentages of proteins involved in each molecular function and found in each cellular location are indicated in brackets.

Using the UniProt database [19], we determined the subcellular locations and functions (or biological processes) of each of the 59 proteins identified by MS analysis. The original spot for each protein, the spot fold-increase or fold-decrease in one cell line versus another cell line, the protein name, the molecular mass, the isoelectric point, the number of unique peptides allowing the protein identification in the MS analysis, and the UniProt accession number of the protein are listed (Table 1; see Additional file 2 for additional data). The information about the molecular function and/or biological process was found for most proteins. Important proteins involved in cell proliferation were differentially expressed following 17β-HSD1 overexpression. These proteins include PCNA, peroxiredoxin 2, BRCA2 and CDKN1A interacting protein (BCCIP) and ribonuclease/angiogenin inhibitor 1 (RNH1). Intriguingly, the metastasis inhibition factor nm23 (nm23-H1), an enzyme known to act as a downregulator of breast cancer metastasis, was upregulated by 17β-HSD1 and found in a spot unique to MCF7-17βHSD1 as compared to MCF7. The repartitions of the functions and subcellular locations of the 59 differentially expressed proteins are illustrated and the percentages of proteins involved in each molecular function and found in each cellular location are indicated (Figure 2). Overexpression of 17β-HSD1 in MCF7 cells causes a differential expression of proteins that act mainly in metabolism (5.5% upregulated protein and 5.5% repressed), mRNA processing (3.1% induced and 7.9% repressed), protein biosynthesis (9%) and transport (8%). Differentially expressed proteins are mainly located in the cytoplasm and nucleus.

**Table 1:** Mass spectrometry identification of proteins differentially expressed between wild type MCF7 cells and MCF7 cells stably transfected with 17β-HSD1 (MCF7-17βHSD1).

Spot	FC	Description	UniProt number	MW exp/ pred	pI exp	Pep	Function and/or biological process
**Spot downregulated in MCF7-17βHSD1 as compared to WT MCF7**							
4183	2.7	Cathepsin D	P07339	28/45	5.0	20	Proteolysis, pathogenesis of diseases (breast cancer)
		Ezrin-radixin-moesin-binding phosphoprotein 50	O14745	58/39	5.3	17	Wnt signaling pathway
3252	2.3	Neudesin	Q9UMX5	19/19	4.7	2	Neuronal differentiation and proliferation
1703	2.2	Ribonuclease/angiogenin inhibitor 1 (RNH1)_(3177)_^a^	P13489	58/50	4.6	24	Regulation of angiogenesis, mRNA catabolism
		BRCA2 and CDKN1A interacting protein (BCCIP)^a^	Q9P287	58/36	4.6	4	Promote cell cycle arrest
		Cell division cycle protein 123 homolog	O75794	58/39	4.6	2	Required for S phase entry of the cell cycle

**Spot unique to WT MCF7**							
2617	U	Poly(rC)-binding protein 2	Q15366	32/39	6.4	11	RNA binding
		Purine nucleoside phosphorylase	P00491	32/32	6.4	6	DNA modification
		BTB/POZ domain-containing protein KCTD15	Q96SI1	32/32	6.4	4	Potassium ion transport
		RING finger protein 114	Q9Y508	32/26	6.4	2	Cell differentiation
4335	U	Peptidyl-prolyl cis-trans isomerase E	Q9UNP9	35/33	5.4	10	Protein folding, mRNA splicing
		Transgelin-2	P37802	20/22	5.6	8	Muscle development
		Splicing factor, arginine/serine-rich 2	Q01130	35/25	5.4	2	mRNA processing
3039	U	RAB11B protein	A5YM50	24/25	5.7	11	Protein transport, signal transduction
		Peroxiredoxin-2^a^	P32119	24/22	5.7	6	Cell redox regulation, anti- apoptosis
		Splicing factor, arginine/serine-rich 3	P84103	24/19	5.7	2	RNA processing in relation with cell proliferation

**Spot upregulated in MCF7-17ΒHSD1 as compared to WT MCF7**							
2714	2.5	Exosome complex exonuclease RRP41	Q9NPD3	29/26	6.2	3	rRNA processing
		Enoyl-CoA hydratase, mitochondrial	P30084	29/31	6.2	6	Fatty acid and lipid metabolism
		Heat shock 70 kDa protein 1	P08107	29/70	6.2	5	Stress response
		Eukaryotic translation initiation factor 4H	Q15056	29/27	6.2	4	Host-virus interaction, protein biosynthesis
2496	4.9	Proliferating cell nuclear antigen (PCNA)^a^	P12004	32/29	4.6	16	DNA replication
5474	4.0	Myosin regulatory light chain 2, nonsarcomeric	P19105	20/20	4.7	5	Cytokinesis, receptor capping, cell locomotion
2300	3.3	60S acidic ribosomal protein P0_(2305)_	A8K4Z4	40/34	5.6	6	Ribosome biogenesis, translation elongation

**Spot unique to MCF7-17ΒHSD1**							
2305	U	17β-hydroxysteroid dehydrogenase type 1_(2300)_	P14061	41/35	5.4	22	Steroid biosynthesis
3177	U	Metastasis inhibition factor nm23 (nm23-H1)^a^	P15531	21/17	5.9	6	Cell cycle and proliferation, nucleotide metabolism
4667	U	60S ribosomal protein L11	P62913	21/20	5.9	2	Binds to 5S ribosomal RNA
4678	U	S-phase kinase-associated protein 1 (SKP1)^a^	P63208	20/19	4.6	2	Ubl conjugation pathway

The function and/or biological process were obtained from the UniProt database [19].

Spot, spot number; FC, fold change; U, unique; MW, molecular weight (kDa); pI exp, isoelectric point as determined from the two-dimensional gel experiments; Pep, number of unique peptides. ^a^Proteins used for RT-qPCR validation. The number after the protein name indicates the additional spot in which the protein was found.

**Figure 3:**
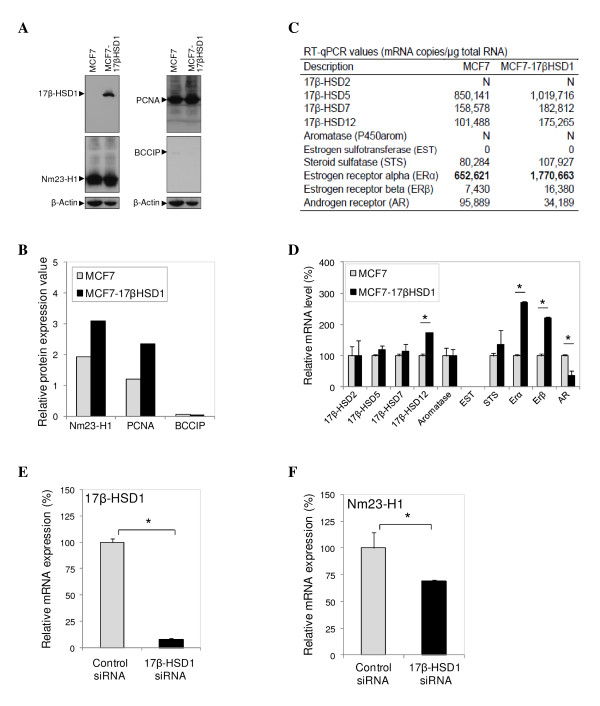
**mRNA and protein level modulation by 17beta-hydroxysteroid dehydrogenase type 1 (17β-HSD1)**. **(A) **Expression of 17β-HSD1, proliferating cell nuclear antigen (PCNA), nm23-H1, and BRCA2 and CDKN1A interacting protein (BCCIP) in wild type (WT) MCF7 cells and MCF7 cells stably transfected with 17β-HSD1 (MCF7-17βHSD1) revealed by western blots. β-actin protein amount was used as internal control. The arrows show the positions of the protein bands. **(B) **The western blot bands in **(A) **were quantified, and the ratio between the protein signals of interest and the β-actin signal was calculated to determine the relative protein expression values for WT MCF7 and MCF7-17βHSD1. **(C) **Reverse transcription quantitative real-time polymerase chain reaction (RT-qPCR) values (mRNA copies/µg total RNA) of mRNAs encoding proteins involved in estradiol production and/or action in WT MCF7 and MCF7-17βHSD1. N, negligible (RT-qPCR values < 1,000); 0, mRNA not detected after many rounds of amplification. **(D) **Relative mRNA expression values of enzymes involved in estradiol production in MCF7-17βHSD1 as compared to WT MCF7. The mRNA levels in WT MCF7 cells were fixed at 100. **(E) **and **(F) **Relative 17β-HSD1 **(E) **and nm23-H1 **(F) **mRNA expression in siRNA-transfected T47D cells. T47D cells were transfected with 17β-HSD1 siRNA or control siRNA and 17β-HSD1 mRNA was quantified by RT-qPCR. mRNA quantity in control-siRNA transfected cells was fixed at 100. Error bars represent standard deviation. *P **<**0.05 analyzed by Student's *t-*test.

In order to verify the differential expression of individual proteins in MCF7 and MCF7-17βHSD1, we performed western blot analysis on total protein extracts for three of the identified proteins, PCNA, nm23-H1 and BCCIP. The differential expression of PCNA and nm23-H1 in MCF7 and MCF7-17βHSD1 was confirmed. However, BCCIP barely exhibited a protein band and thus, the observed relative protein expression values in the two cell lines should be considered with caution (Figure 3A and 3B).

### The mRNA levels of enzymes involved in cell proliferation are regulated by 17β-HSD1

Next, we investigated if 17β-HSD1 influences the transcription of genes involved in cell proliferation. To do this, six proteins involved in cell proliferation, cancerogenesis or metastasis regulation were selected: PCNA, peroxiredoxin 2, nm23-H1, S-phase kinase-associated protein 1 (SKP1), BCCIP and RNH1. Their mRNA levels were measured by RT-qPCR analysis of total RNA extracts from MCF7 and MCF7-17βHSD1 cell lines (Table 2). The increases in mRNA levels of PCNA, peroxiredoxin 2, nm23-H1 and BCCIP following 17β-HSD1 overexpression were significant (more than 2-fold). The most regulated mRNA is that of nm23-H1 which was 3.6-fold higher in MCF7-17βHSD1 cells than in MCF7 cells (Table 2).

**Table 2:** mRNA quantification by RT-qPCR of genes involved in breast cancer cell proliferation within wild type MCF7 and MCF7 cells stably transfected with 17β-HSD1 (MCF7-17βHSD1) and comparison with two-dimensional gel data.

**Description**^a^	MCF7	MCF7-17βHSD1	Fold **regulation**^b^	Correlation 2-D gel and RT-qPCR
RT-qPCR value (mRNA copies/µg total RNA)				
Proliferating cell nuclear antigen (PCNA)	1,599,813	4,483,982	+ 2.8	Yes^c^
Peroxiredoxin 2	4,078,760	8,585,424	+ 2.1	No^c^
Metastasis inhibition factor nm23 (nm23-H1)	5,366,763	19,356,416	+ 3.6	Yes^c^
S-phase kinase-associated protein 1 (SKP1)	3,810,452	5,714,509	+ 1.5	Yes
BRCA2 and CDKN1A interacting protein (BCCIP)	345,839	1,074,647	+ 3.1	No^c^
Ribonuclease/angiogenin inhibitor 1 (RNH1)	1,015,193	727,425	- 1.4	Yes^c^

Standard deviations were < 10% of duplicates.

^a^Proteins were selected for reverse transcription quantitative real-time polymerase chain reaction (RT-qPCR) after their identification by mass spectrometry analysis of two-dimensional (2-D) gel protein spots.

^b^Fold regulation of mRNA levels in MCF7 cells stably transfected with 17β-HSD1 (MCF7-17βHSD1) compared to wild type MCF7 cells; +, fold increase; -, fold decrease).

^c^Mass spectrometry analysis showed that the two-dimensional spot contained several proteins including the indicated protein.

We further evaluated the correlation between mRNA and protein levels by comparing data from the RT-qPCR and the proteomic analyses. Proteomics and RT-qPCR data were considered to correlate if the mRNA level and protein spots were regulated in the same direction. It must be noted that the observed correlations are semiquantitative, since two-dimensional gel data are considered semiquantitative, and some spots contained more than one protein. In addition, some proteins were found in several spots, which can be the effect of post-translational modifications [20]. When comparing MCF7-17βHSD1 to MCF7, we found that RNH1, a regulator of angiogenesis, was downregulated at both protein and transcript levels, whereas PCNA, SKP1 and nm23-H1 were upregulated at both protein and transcript levels. With the exception of peroxiredoxin-2 (which is an anti-apoptosis protein) and BCCIP (a promoter of cell cycle arrest), all the other four proteins for which the mRNA expression was evaluated exhibited regulation in the same direction for protein and mRNA in MCF7-17βHSD1 as compared to MCF7 (Table 2). These data can indicate the existence of a semiquantitative correlation between protein and mRNA expression. Thus, it may be possible to predict the presence of a protein based on its gene expression or inversely. However, as suggested in a previous study [21], the correlation between mRNA and protein levels may not be sufficient to predict protein expression levels from quantitative mRNA data.

### Transcription of various genes involved in E2 production

Because 17β-HSD1 is a pivotal enzyme in the synthesis of E2, a hormonal steroid playing a major role in breast cancer induction and progression, we were interested to know if its overexpression in MCF7 cells would influence the expression of other genes involved in the hormone synthesis, inactivation and action. The mRNA levels of these proteins, which include 17β-HSDs type 2 (17β-HSD2), type 5 (17β-HSD5), type 7 (17β-HSD7), type 12 (17β-HSD12), aromatase (or P450arom), estrogen sulfotransferase (EST), STS, androgen receptor (AR), estrogen receptor alpha (ERα) and estrogen receptor beta (ERβ), were quantified by RT-qPCR (Figure 3C). RT-qPCR analyses revealed that the overexpression of 17β-HSD1 in MCF7 cells induces an increase in the mRNA expression of 17β-HSD5, STS, 17β-HSD12 and ERβ by 20, 33, 73 and 120%, respectively, while inhibiting AR expression by 64%. The highest mRNA-level modulation was observed with ERα, which exhibited a significant increase of 171%. The increase in 17β-HSD7 was small, whereas no modulation was observed with 17β-HSD2 and aromatase expression (Figure 3C and 3D). These results show that the expression of 17β-HSD1 can influence that of other genes implicated in estradiol metabolism and action, especially the estrogen receptors (ER), and further confirm the enzyme role in producing active estrogen and inactivation of DHT [11].

### Correlation between 17β-HSD1 and nm23-H1 expression

Since our proteomic and RT-qPCR data showed that 17β-HSD1 overexpression increases the metastasis inhibition factor nm23-H1 mRNA and protein levels, we were interested to know if 17β-HSD1 knockdown would decrease nm23-H1 expression. T47D cells were chosen for this investigation because the cell line expresses a high level of endogenous 17β-HSD1 [11,15,22]. Cells were transfected with 17β-HSD1-specific siRNA and with scramble siRNA (control siRNA) and total RNA was extracted 48 hours after transfection. The 17β-HSD1 and nm23-H1 mRNAs were quantified by RT-qPCR. The efficacy of 17β-HSD1 knockdown by its specific siRNA was demonstrated since 92% inhibition of 17β-HSD1 mRNA was observed (Figure 3E). Nm23-H1 mRNA levels were compared in control-siRNA- and 17β-HSD1-siRNA-transfected T47D cells. A decrease of 31% of nm23-H1 mRNA expression was observed after 17β-HSD1 gene knockdown (Figure 3F). These results, combined with proteomic and RT-qPCR analyses of MCF7 and MCF7-17βHSD1, indicate a positive correlation between 17β-HSD1 and nm23-H1 expression.

### Regulation of cell migration by 17β-HSD1

**Figure 4:**
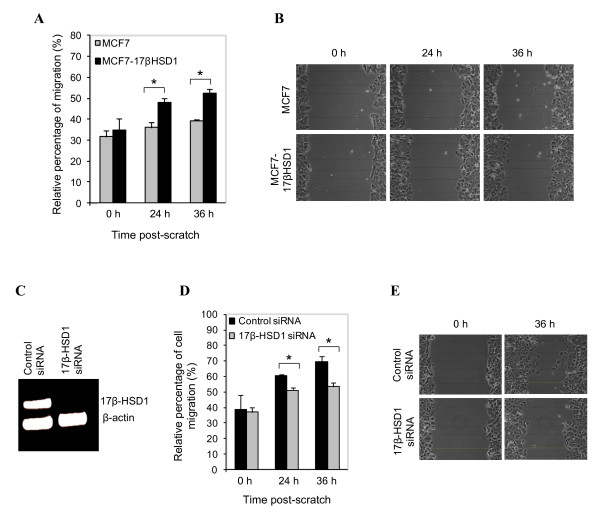
**17beta-hydroxysteroid dehydrogenase type 1 **(**17β-HSD1) is a positive regulator of MCF7 cell migration**. **(A) **and (**B) **Comparison of cell migration between wild type (WT) MCF7 and MCF7 cells stably transfected with 17β-HSD1 (MCF7-17βHSD1). A scratch was applied to WT MCF7 and MCF7-17βHSD1 cells confluent in dishes (3.5 cm diameter) and the ability to invade the scratch was measured. **(A) **The relative migration of WT MCF7 and MCF7-17βHSD1 cells at 24 and 36 hours post-scratch was quantified. The scratch widths, two near the border and three in the middle of the scratch (as shown in **B**) were measured at the indicated time points using the NIH ImageJ software and data were used to calculate the percentage of migration. **(B) **Results showed that WT MCF7 cells have less ability to invade the scratch than the MCF7-17βHSD1 cells. Lines represent measurements made to assess modifications in scratch widths. **(C) **17β-HSD1 knockdown by siRNA in MCF7-17βHSD1 cells. Semiquantitative reverse transcription polymerase chain reaction (RT-PCR) was performed using 17β-HSD1 and β-actin primers and total RNA extracted from MCF7-17βHSD1 cells transfected with 17β-HSD1-specific siRNAs or control siRNA. **(D) **and **(E) **Effect of 17β-HSD1 knockdown on MCF7-17βHSD1 cell migration. MCF7-17βHSD1 cells were transfected with 17β-HSD1-specific siRNAs or control siRNA for 48 hours before creating a wound by scraping the cell monolayer. Cells transfected with 17β-HSD1 siRNA have less ability to invade the scratch than cells transfected with control siRNA. All experiments were done in quadruplicate, and representative images of cell progression in the scratch are shown. Error bars represent standard deviation. *P **<**0.05 analyzed by Student's *t*-test.

To investigate if 17β-HSD1 could have any implication in cancer metastasis, we evaluated the effect of its expression on MCF7 cell migration by means of a wound-healing assay. The migrations of WT MCF7 and MCF7-17βHSD1 cells were first compared. We found that cell migration was higher in MCF7-17βHSD1 stably overexpressing 17β-HSD1 than in WT MCF7 cells, showing that MCF7-17βHSD1 cells have more ability to invade a scratch than WT MCF7 cells (Figure 4A and 4B). The effect of 17β-HSD1 knockdown on cell migration was then tested in MCF7-17βHSD1 cells transfected with 17β-HSD1-specific siRNAs or control siRNA. Semiquantitative RT-PCR analysis showed a 98% decrease of 17β-HSD1 mRNA after transfection of MCF7-17βHSD1 cells with specific siRNAs as compared to the control cells (Figure 4C). Importantly, the knockdown of 17β-HSD1 in MCF7-17βHSD1 cells was associated with a decreased cell migration (by 16.1%, *P *< 0.05) compared to cells transfected with control siRNA (Figure 4D and 4E). Taken together, these results show that 17β-HSD1 expression is positively correlated with MCF7 cell migration.

## Discussion

### Proteomic modifications of MCF7 cells in response to 17β-HSD1 overexpression

In a previous study, we showed that modulating the expression of the steroid-converting enzyme 17β-HSD1 in MCF7 and T47D cells led to a differential cell growth compared to the parent cells, cultured in medium containing E2 [11]. The present study compared the proteomes of the stably transfected MCF7-17βHSD1 and WT MCF7 cells and established the first differential profile of a cell line overexpressing the enzyme and its parent cell. Our proteomics data revealed that increasing 17β-HSD1 expression significantly modulates the expression of proteins involved in various functional activities such as cell cycle, cell growth, apoptosis and carcinogenesis. Examples include PCNA, BCCIP and peroxiredoxin-2. Considering the functions of these enzymes in breast cancer, the directions in which their expression is regulated by 17β-HSD1 agree with its role in increasing breast cancer cell growth. This can reveal the factors that make 17β-HSD1 stably-transfected MCF7 cells grow faster than the WT MCF7 cells when cultured in medium containing E2 [11]. The four most represented functional activities for the modulated proteins are metabolism (examples include alpha-N-acetylgalactosaminidase and mitochondrial enoyl-CoA hydratase), mRNA processing (eukaryotic initiation factor 4A-III and arginine/serine-rich splicing factor 2), protein biosynthesis (elongation factor 1-gamma) and transport (endoplasmic reticulum resident protein ERp29 and RAB11B protein) (Figure 2, Table 1 and Additional file 2). The predominant locations of differentially expressed proteins in the nucleus and cytoplasm might reflect their functions in mRNA processing and protein biosynthesis. These four functions are known to be essential in steroid signalling which involves fast nongenomic activities (including the transport and metabolism of signalling molecules) and genomic mechanisms mediated by their specific receptors; these later mechanisms comprise gene transcription (RNA formation and mRNA processing) and protein biosynthesis [14] related to cell growth and regulation. The presence of E2 in MCF7 and MCF7-17βHSD cell culture medium and the change in expression of a large number of proteins involved in these four functions following 17β-HSD1 overexpression suggest a modulation of E2 effects by 17β-HSD1. In fact, 17β-HSD enzymes fit well into the concept of pre-receptor regulation of steroid action as they efficiently alter the binding of steroids to their genomic and nongenomic receptors and effectors, acting as a metabolic switch prior to the function of these receptors [14]. The concept of pre-receptor regulation of E2 action by 17β-HSD1 corroborates with its effect on the modulation of E2 responsiveness of pS2 genes in T47D cells [11], since E2 exerts its biological effect on breast cancer predominantly via the mediation of ERα and ERβ [23,24]. The strong stimulation of ERα (171% increase) and ERβ (120% increase) gene expression and the protein regulation of a large number of non-estrogen-responsive genes caused by 17β-HSD1 overexpression further suggest that the ligand-independent transcriptions of ER target genes are also modulated by the enzyme. Indeed, this stimulation can influence the regulation of gene transcription by ER.

A recent study showed a high level of 17β-HSD2 in the WT MCF7 cell line [25]. Our data, on the contrary, showed negligible expression of 17β-HSD2 in this cell line, in conformity with other studies [15,26]. While 17β-HSD1 has no effect on the expression of the E2-inactivating enzyme 17β-HSD2, it increases the mRNA levels of the E2-producing enzymes 17β-HSDs type 5, 7 and 12, with type 12 having a significant, and the highest increase. This suggests a concerted action of reductive 17β-HSDs to accelerate the cellular E2 biosynthesis.

### Breast cancer cell migration is increased by 17β-HSD1 despite a positive correlation with the metastasis suppressor gene nm23-H1

Two estrogen-responsive genes involved in metastasis regulation, cathepsin D (Table 1) [27] and nm23-H1 [28-30], were found to be differentially expressed at the protein levels following 17β-HSD1 overexpression. Cathepsin D, an independent marker of poor prognosis in breast cancer that correlates with the incidence of clinical metastasis [31], was downregulated. Nm23-H1 was upregulated at the protein level, its mRNA increased 3.6-fold with 17β-HSD1 overexpression (in MCF7-17βHSD1), and its gene expression decreased by 31% following 17β-HSD1 knockdown in T47D. These results demonstrate that 17β-HSD1 expression is positively and closely correlated to nm23-H1 expression. The downregulation of cathepsin D can be related to the increase of nm23-H1 as their negative correlation has already been demonstrated [27]. Patients with malignant melanoma who develop metastases during the first two years after diagnosis have significantly lower levels of tumor nm23-H1 expression (56% of the mean value) compared to patients with less aggressive disease (164%) [32]. The nm23-H1 gene, *NM23*, is known to function as a tumor metastasis suppressor gene and its transcript level is reduced in highly metastatic cells [32,33]. It has been reported that nm23-H1 inhibits cell migration and cancer metastasis by modulating the activity of Rho-family small GTPase enzymes, which are known to play a key role in the actin cytoskeleton dynamics required for cancer cell migration and invasion [34]. In agreement with the strong enhancement of nm23-H1 mRNA level by 17β-HSD1, we hypothesized the implication of 17β-HSD1 in tumor metastasis. Until now, quantitative analyses of the transcripts of estrogen-producing enzymes in breast cancer metastases have not demonstrated any significant association between 17β-HSD1 mRNA level and metastases, although the sulfatase and aromatase mRNA levels were significantly associated with the presence of metastases in some studies [35-37]. Using the wound-healing assay, we demonstrated for the first time that increasing 17β-HSD1 expression led to the increase of MCF7 cell migration while 17β-HSD1 knockdown decreased MCF7 cell migration. Our study thus shows for the first time that 17β-HSD1 expression is positively correlated with the migration of the breast cancer cell line MCF7, revealing its role as a positive regulator of cell migration, contrary to nm23-H1.

E2-induced time-dependent increases in the abundance of nm23-H1 mRNA and protein are coincident with the expression level of its receptor ERα [29], which has been shown to interact with the non-metastasis gene nm23-H1 [27,29]. Since our cell models were cultivated in the presence of E2 and our data showed that 17β-HSD1 positively regulates ERα mRNA level, one could postulate that the positive correlation between nm23-H1 and 17β-HSD1 expression is coincident with the activation of nm23-H1 expression by ERα and E2, which are increased with 17β-HSD1 expression. Thus, 17β-HSD1 may indirectly affect nm23-H1 expression via ERα action. On the other hand, the increase of MCF7 cell migration by 17β-HSD1, demonstrated in the present study, corroborates with its role in stimulating breast cancer cell growth [11] and the poor prognosis for patients in whom 17β-HSD1 is highly expressed in the breast [13]. This may open a new study on the role of this multifunctional steroid enzyme, that appeared early in evolution [38,39], revealing a complex mechanism in breast cancer with its expression. The latter may involve protein-protein and protein-DNA interactions among ERα, *NM23*, 17β-HSD1, AR, cathepsin D and other genes and proteins. The role of 17β-HSD1 is in keeping with evidence from recent studies [22,40].

## Conclusions

Our study demonstrates that 17β-HSD1 affects breast cancer cell proteome and modulates expression of several genes at both mRNA and protein levels. Among the individual mRNA and proteins for which the regulation was investigated, the most strongly modulated by 17β-HSD1 are ERα and nm23-H1. Intriguing observations are that although 17β-HSD1 strongly stimulates nm23-H1 expression, it is associated with an increased MCF7 cell migration. Here, we report the general study of proteomics with 17β-HSD1 expression modification, while the mechanism on cell migration modification opens a new study of interest for additional roles of the well-known steroid-converting enzyme. It can be of great interest to investigate 17β-HSD1 role in cancer metastasis formation.

## Abbreviations

AR: androgen receptor; BCCIP: BRCA2 and CDKN1A interacting protein; 17β-HSD1: 17beta-hydroxysteroid dehydrogenase type 1; CHAPS: 3-[(3-cholamidopropyl)dimethylammonio]-1-propanesulfonate; cm: centimeter; cm^2^: square centimeter; DHEA: dehydroepiandrosterone; DHEA-S: dehydroepiandrosterone sulfate; DHT: dihydrotestosterone; E1: estrone; E1S: estrone sulphate; E2: estradiol; ER: estrogen receptor; ERα: estrogen receptor alpha; ERβ: estrogen receptor beta; ES-MS/MS: electrospray tandem mass spectrometry; EST: estrogen sulfotransferase; FBS: fetal bovine serum; IPG: immobilized pH gradient; INCA: Intelligent Noise Correction Algorithm; MS: mass spectrometry; nano LC: nanoscale capillary liquid chromatography; NADPH: the reduced form of nicotinamide adenine dinucleotide phosphate; PBS: phosphate-buffered saline; PBS-T: PBS-tween 20; PCNA: proliferating cell nuclear antigen; PMSF: phenylmethylsulfonyl fluoride; RIN: RNA integrity number; RNH1: ribonuclease/angiogenin inhibitor 1; RP: reversed-phase; RT-PCR: reverse transcription polymerase chain reaction; RT-qPCR: reverse transcription quantitative real-time polymerase chain reaction; SDS-PAGE: sodium dodecyl sulphate-polyacrylamide gel electrophoresis; SKP1: S-phase kinase-associated protein 1; STS: steroid sulfatase; WT: wild type.

## Competing interests

The authors declare that they have no competing interests.

## Authors' contributions

JAA, MZ and FH carried out the experimental studies. JAA and SXL designed the study. JAA, JH and SXL prepared the manuscript. All authors read and approved the final manuscript.

## Supplementary Material

Additional file 1 **Table showing the primers used for reverse transcription quantitative real-time polymerase chain reaction**. Click here for file

Additional file 2 **Table of additional data for mass spectrometry identification of proteins differentially expressed between wild type MCF7 and MCF7 stably transfected with 17β-HSD1 (MCF7-17βHSD1)**. Click here for file
