# Supplementary material for: 17beta-hydroxysteroid dehydrogenase type 1 modulates breast cancer protein profile and impacts cell migration
Source: Breast Cancer Res. 2012 Jun 12;14(3):R92. doi: 10.1186/bcr3207 (PMC3446355; doi:10.1186/bcr3207)
Supplement: Additional file 1 — Table showing the primers used for reverse transcription quantitative real-time polymerase chain reaction. [file bcr3207-S1.DOCX]

| **Additional file 1, Table S1.** Primers used for reverse transcription quantitative real-time PCR. | | | |
| --- | --- | --- | --- |
| Gene symbol | GenBank number (NCBI) | Primer sequence (5' → 3') Forword/reverse | cDNA fragment^a^ |
|  |  |  |  |
| *PCNA* | NM_002592 | gggcttcgacacctaccgctg/ctttctcctggtttggtgcttca | 404-570 |
| *PRDX2* | NM_005809 | gcccacgcagctttcagtcat/agtccgacagcttcacctctttg | 132-247 |
| ***NME1*** *(NDKA)* | NM_198175 | tgtggagagtgcagagaaggaga/gaaggaggggaaatggatgtga | 694-833 |
| *SKP1* | NM_006930 | tgcacccaccacaaggatgac/catttttgatattgaaggtcttgcgaa | 363-609 |
| *BCCIP* | NM_016567 | agctggacaagtttttaaatgacacc/tcctgcttccacaaatgtcttactaat | 514-713 |
| *RNH1* | NM_002939 | ctgtggatctgggagtgtggc/caggacttcacccacagcgactc | 1254-1441 |
| *HSD17B2* | NM_002153 | gcgcctctcggtgctccaaatg/cggccatgcattgtttgtagtcagtca | 557-738 |
| *AKR1C3* | NM_003739 | caaccaggtagaatgtcatccgtat/acccatcgtttgtctcgttga | 633-752 |
| *HSD17B7* | NM_016371 | tccaccaaaagcctgaatctctc/gggctcactatgtttctcaggc | 826-1118 |
| *HSD17B12* | NM_016142 | ggctggtcttgaaatcggcat/tgccactgccagatgaaatgtt | 439-650 |
| *CYP19A1* | M28420 | cgacaggctggtaccgcatgctc/aagaggcaataataaaggaaatccagac | 734-856 |
| *SULT1E1* | NM_005420 | aagcgttccaggcaagaccagatg/tttgcacttttccacatcaccctctt | 205-323 |
| *STS (ARSC1)* | M16505 | agccctaatcctgacccttttcttgg/ccgcctccaccgttagcctct | 889-1025 |
| *ESR1* | NM_000125 | tgcaaaatctaacccctaaggaagtg/ctcccagtacccacagtccatctc | 5258-5550 |
| *ESR2a* | NM_001437 | cgccgtgaccgatgctttgg/gcccttgttactcgcatgcctgac | 1711-1834 |
| *AR* | NM_000044 | agccattgagccaggtgtagtgt/catcctggagttgacattggtgaa | 3152-3401 |
| ^a^Downstream position from the ATG start codon. | | | |
