# Supplementary material for: 17beta-hydroxysteroid dehydrogenase type 1 modulates breast cancer protein profile and impacts cell migration
Source: Breast Cancer Res. 2012 Jun 12;14(3):R92. doi: 10.1186/bcr3207 (PMC3446355; doi:10.1186/bcr3207)
Supplement: Additional file 2 — Table of additional data for mass spectrometry identification of proteins differentially expressed between wild type MCF7 and MCF7 stably transfected with 17β-HSD1 (MCF7-17βHSD1). [file bcr3207-S2.DOCX]

| **Additional file 2, Table S2.** Additional data of mass spectrometry identification of proteins differentially expressed between wild type MCF7 and MCF7 stably transfected with 17β-HSD1 (MCF7-17βHSD1).  The function and/or biological process were from the UniProt database [19].  Spot, spot number; FC, fold change; U, unique; MW, molecular weight (kDa); pI exp, isoelectric point as determined from the 2-D gel experiments; Pep, number of unique peptides. The number after the protein name indicated the additional spot in which the protein was found. | | | | | | | |
| --- | --- | --- | --- | --- | --- | --- | --- |
|  | | | | | | | |
| Spot | FC | Description | UniProt number | MW exp/  pred | pI  exp | Pep | Function and/or biological process |
| SPOT DOWN-REGULATED IN MCF7-17βHSD1 AS COMPARED TO WT MCF7 | | | | | | | |
| 4183 | 2.7 | Keratin, type II cytoskeletal 8_(2196; 3499)_ | P05787 | 58/54 | 5.3 | 60 | [Host-virus interaction](http://www.uniprot.org/keywords/KW-0945) |
| 3252 | 2.3 | Calpain small subunit 1 | P04632 | 19/28 | 4.7 | 6 | Proteolyse catalysis |
| 2214 | 3.0 | Keratin, type I cytoskeletal 19_(2196)_ | P08727 | 44/44 | 4.6 | 45 | Myofiber organization, [host-virus interaction](http://www.uniprot.org/keywords/KW-0945) |
| 2196 | 2.6 | cDNA FLJ75154, highly similar to Homo sapiens heterogeneous nuclear ribonucleoprotein C (C1/C2), mRNA | A8K9A4 | 44/34 | 4.8 | 3 | Nucleic acid binding |
| 1703 | 2.2 | Alpha-N-acetylgalactosaminidase | P17050 | 58/47 | 4.6 | 5 | [Carbohydrate metabolic process](http://www.ebi.ac.uk/ego/DisplayGoTerm?id=GO:0005975) |
|  |  | Elongation factor 1-gamma | P26641 | 58/50 | 4.6 | 3 | [Protein biosynthesis](http://www.uniprot.org/keywords/KW-0648) |
| 1957 | 5.4 | Heterogeneous nuclear ribonucleoprotein H | P31943 | 51/49 | 6.3 | 11 | [mRNA processing](http://www.uniprot.org/keywords/KW-0507) |
|  |  | cDNA, FLJ92536, highly similar to Homo sapiens isocitrate dehydrogenase 1 (NADP+), soluble (IDH1), mRNA | B2R5M8 | 51/47 | 6.3 | 3 | Glutathione and [isocitrate metabolic process](http://www.ebi.ac.uk/ego/DisplayGoTerm?id=GO:0006102) |
|  |  | COP9 signalosome complex subunit 3 | Q9UNS2 | 51/48 | 6.3 | 2 | Involved in various cellular and developmental processes |
|  |  | Actin, cytoplasmic 1_(2305)_ | P60709 | 47/42 | 4.5 | 7 | Cell motility |
|  |  | Phosphoglycerate kinase 1 | P00558 | 51/45 | 6.3 | 2 | [Glycolysis](http://www.uniprot.org/keywords/KW-0324) |
| SPOT UNIQUE TO WT MCF7 | | | | | | | |
| 4335 | U | Peptidyl-prolyl cis-trans isomerase E | Q9UNP9 | 35/33 | 5.4 | 10 | Protein folding, [mRNA splicing](http://www.uniprot.org/keywords/KW-0508) |
|  |  | Uncharacterized protein SFRS7 (Splicing factor, arginine/serine-rich 7, 35kDa, isoform CRA_a) | A6NNE8 | 35/16 | 5.4 | 2 | Unknown |
|  |  | Palmitoyl-protein thioesterase 1 | P50897 | 35/34 | 5.4 | 2 | [Sensory transduction](http://www.uniprot.org/keywords/KW-0716) |
| SPOT UP-REGULATED IN MCF7-17βHSD1 AS COMPARED TO WT MCF7 | | | | | | | |
| 2496 | 4.9 | UPF0368 protein Cxorf26 | Q9BVG4 | 32/26 | 4.6 | 2 | Unknown |
| 2714 | 2.5 | BTB/POZ domain-containing protein KCTD21 | Q4G0X4 | 29/30 | 6.2 | 5 | [Potassium ion transport](http://www.ebi.ac.uk/ego/DisplayGoTerm?id=GO:0006813) |
|  |  | Endoplasmic reticulum protein ERp29 | P30040 | 29/29 | 6.2 | 4 | [Protein transport](http://www.ebi.ac.uk/ego/DisplayGoTerm?id=GO:0006886), [folding](http://www.ebi.ac.uk/ego/DisplayGoTerm?id=GO:0006457) and [secretion](http://www.ebi.ac.uk/ego/DisplayGoTerm?id=GO:0009306) |
|  |  | Haloacid dehalogenase-like hydrolase domain-containing protein 3 | Q9BSH5 | 29/28 | 6.2 | 4 | [Metabolic process](http://www.ebi.ac.uk/ego/DisplayGoTerm?id=GO:0008152) |
|  |  | High-mobility group box 1 | A5D8W9 | 29/25 | 6.2 | 4 | DNA bending |
| SPOT UNIQUE TO MCF7-17βHSD1 | | | | | | | |
| 2305 | U | UPF0553 protein C9orf64 | Q5T6V5 | 41/39 | 5.4 | 2 | Unknown |
|  |  |  |  |  |  |  |  |
